# Development of a Variable-Configuration Bionic Robotic Fish

**DOI:** 10.3390/biomimetics8050407

**Published:** 2023-09-01

**Authors:** Dan Xia, Yuyao Li, Zhihan Li, Mengqian Tian, Xingsong Wang

**Affiliations:** School of Mechanical Engineering, Southeast University, Nanjing 210096, China; l598658625@163.com (Y.L.); lzh809963603@163.com (Z.L.); xswang@seu.edu.cn (X.W.)

**Keywords:** bionic robotic fish, variable configurations, flexible fins, pectoral fin slap propulsion, tail fin swing propulsion

## Abstract

Bionic robotic fish have advantages over traditional underwater propulsion. Most of the existing studies have been conducted with only one type of fish as a bionic object, but a single propulsion mode may not be able to achieve the different needs of underwater operations. In this paper, we designed a pneumatic variable-configuration soft bionic fish and completed the overall structure design. It was built with a cownose ray as the main-configuration bionic object and a *Caranx melampygus* as the secondary-configuration bionic object. The base structure, actuators, and variable-configuration modules of the robot were made using flexible materials. After completing the design of the structure and control system of the robot, the prototype was manufactured and an underwater test was completed. The tests results indicated that the robot fish could achieve underwater linear propulsion and turning movements in both configurations. The maximum propulsion speed of the main configuration was 38.24 mm/s and the turning angle speed was 5.6°/s, and the maximum propulsion speed of its secondary configuration was 43.05 mm/s and the turning angle speed was 30°/s. The feasibility of the machine fish structure and control scheme were verified.

## 1. Introduction

With the continuous development of bionics and the human demand for marine exploitation, research into underwater robots with fish as bionic objects is currently a hot topic [1]. According to the different propulsion methods, existing studies have classified bionic robotic fish into body and/or caudal fin propulsion mode (BCF) and median and/or paired-fin propulsion mode (MPF). The fish in BCF mode can swim and turn fast, swim efficiently, and propel well. The fish in MPF mode swim at a lower speed, but they also perturb the current less, which is excellent in terms of maneuverability and handling [2]. Compared with traditional underwater propulsion, the bionic robotic fish has better swimming efficiency and propulsion performance. The robotic fish also perturbs the underwater environment less. Therefore, it is necessary to study the propulsion principle and swimming performance of robotic fish underwater.

With the continuous development of computer technology, material science, etc., in recent years, many scholars have researched the underwater propulsion ability of bionic fish through the design of fish body structure materials, computational fluid dynamics simulation analysis, and bionic control methods. In order to improve the underwater performance of bionic robotic fish with different propulsion modes by addressing the characteristics of the two different propulsion modes, an innovative and optimized design of the bionic robotic fish is needed. Yan et al., used the fish body wave equation and penalty function to design and optimize a bionic fishtail and designed the SMG-CPG control system to implement the drive [3]. Katzschmann et al. proposed a hydraulically driven machine fish consisting of a rigid head and a flexible tail. The flexible tail is driven by a hydraulic pump at the desired fluctuation frequency and amplitude. The outlet of the hydraulic pump is connected to the flexible body so that the water flows between the two inner chambers in a closed loop. The change in flow direction causes the tail to swing left and right, propelling the robot forward [4,5]. Xia et al., applied highly flexible origami technology to the fishtail, and the hybrid neutral layer of the fishtail was designed according to bionic principles. They improved the bionic fish propulsion performance through this method [6]. Chen et al., designed a hybrid-driven robotic fish with two active joints at the tail. The first joint near the head of the robotic fish is driven by a servo motor and mainly used for propulsion. The second one near the tail is driven by an ionic polymer–metal composite artificial muscle to achieve steering [7]. The bionic parameters and motions of the cownose ray were analyzed by Cai et al. The complex motion deformation of the cownose ray was broken down into two parts, chordwise wave propagation and spreading flutter motion, and the mechanical structure design principles and motion principles of the multi-joint driven fins were proposed [8,9]. Zhang et al., designed a soft robotic fish using a manta ray as the bionic object. They used tough and stiff hydrogels as the structural elements to realize underwater swimming motions, as well as a dielectric elastomer as the actuating unit [10]. Bianchi et al. designed a bioinspired robot mimicking the cownose ray and conducted experimental studies. The flexible pectoral fins are made of silicone rubber. Each of them is actuated by a servo motor driving a link inside the leading edge. Two small rigid caudal fins are present to improve the robot’s maneuverability [11]. Most of the existing studies were conducted to optimize the structural design and performance of machine fish for the characteristics possessed by each of the two propulsion modes, and certain results have been achieved. However, there are fewer relevant studies that can realize two propulsion modes of machine fish simultaneously.

For the complex situation underwater, there are different requirements for the shape, stability, and speed of the machine fish. On the one hand, it is possible to consider the targeted design and research of different propulsion modes according to different underwater operation requirements and develop customized machine fish for different operating conditions. On the other hand, it may be difficult to meet specific operational requirements when a single propulsion mode is used [12,13], and machine fish with multiple propulsion modes to switch between can be developed. Designs have been presented of two propulsion modes for a single bionic robotic fish, and variable configurations are implemented to allow it to switch between the two modes. The machine fish can be adjusted to different conditions underwater to improve its propulsion efficiency and operational performance in a comprehensive manner. Research on such variable-configuration robotic fish in China and abroad has not been reported yet.

In summary, we designed a variable-configuration bionic robotic fish consisting of two configurations. The main structure of the variable-configuration underwater robot is a cownose ray. Based on the shape and structural characteristics of the bionic object, propulsion is achieved through pectoral fin slapping. The secondary structure is a *Caranx melampygus*. The body of the fish changes from horizontal flat to lateral flat, with the broad pectoral fins retracted, and propulsion is achieved through tail swing. A design study of the structure and control system of the machine fish was conducted, and tests on the propulsion performance of both configurations in straight lines and turns underwater were carried out. The feasibility of the design and control method of the machine fish structure was initially determined, which provided a reliable basis for the subsequent simulation study and performance optimization. In addition, the pneumatic robotic fish designed in this paper can provide some reference for research related to underwater propulsion actuators, and its variable-configuration design can also provide a new idea for the development of underwater bionic robotic fish.

## 2. Structural Design of Variable-Configuration Robotic Fish

### 2.1. Structural Design of the Main Configuration of the Cownose Ray

#### 2.1.1. Biological Characteristics of the Cownose Ray

The cownose ray belongs to the order Rayiformes of the general order Chondrichthyes. The entire body of the fish is horizontal flat, with a wide body plate and a rhomboid or diamond shape. Its propulsion mode belongs to the MPF mode. The cownose ray has strong swimming maneuverability, a large pectoral fin loading area, and low swimming disturbance, and it is good at long-distance migratory swimming [14]. The pectoral fin slap propulsion mode is less efficient compared to the tail fin swing propulsion mode, but it is more flexible and better adapted to the complex environment [15,16]. The main configuration of the robotic fish designed in this paper uses a cownose ray as the bionic object and adopts the pectoral fin slap propulsion mode.

#### 2.1.2. Physical Model and Motion Analysis of the Cownose Ray

The cownose ray has several major parts such as head, body, pectoral fin, and tail. The head and tail have a relatively small influence on the propulsive action and are not considered in the design in order to be able to realize the simplicity of the model [17,18]. The fish body part needs to complete the conformation variation. Therefore, we mainly studied the outline of the pectoral fins of cownose rays and established a physical model. A typical Rhinoptera bonasus was selected as the mimic object, and the shape scale parameters of the robotic fish were established as 26.7–30.7 cm for body disc length and 43.8–49.0 cm for body disc width, based on the measurements of Rosenberger et al. [19].

**Figure 1:**
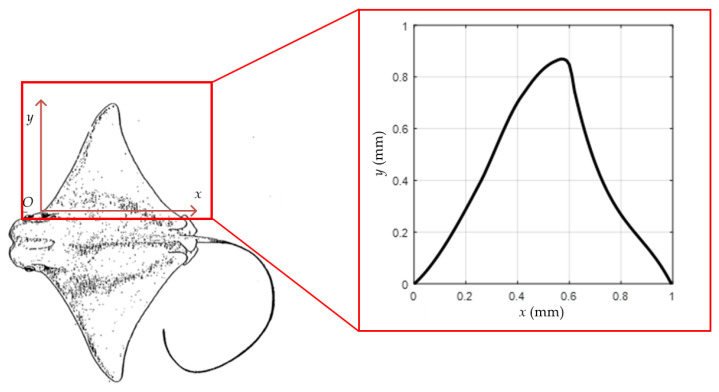
Profile and pectoral fin fitting curve of the cownose ray.

The pectoral fin coordinate system was established on the basis of the profile of the cownose ray measured in the reference [20]. The origin O is the intersection of the front of one pectoral fin with the head. The *x*-axis is the direction from the head to the tail, and the direction is defined as chordal. The *y*-axis is the direction from the trunk to the tip of the pectoral fin, and the *z*-axis is perpendicular to the XOY plane upwards. The coordinate values of a series of points were obtained by extracting the edge contour of its pectoral fin surface. After normalizing the coordinate values, a polynomial function was used to fit them to the segments, and the resulting polynomial segmentation function is shown in Equation (1). The profile of the cownose ray and the fitted curve of the pectoral fin are shown in Figure 1. The curve of the pectoral fin profile on the other side was symmetrical to its midline about the trunk. The physical model of the pectoral fin portion of the cownose ray can be obtained through an isometric magnification of the profile by 200 times.

(1) $$y=\left\{\begin{array}{ll} -4.309x^{3}+3.647x^{2}+0.887x & \left(0<x<0.265\right)\\ -29.666x^{3}+27.951x^{2}-6.454x+0.710 & \left(0.265<x<0.425\right)\\ -8.420x^{3}+7.652x^{2}-0.432x+0.186 & \left(0.425<x<0.545\right)\\ -135.252x^{3}+215.632x^{2}-113.982x+20.797 & \left(0.545<x<0.595\right)\\ -77.128x^{3}+81.189x-24.699 & \left(0.595<x<0.615\right)\\ -16.813x^{3}+44.025x^{2}-39.612x+13.390 & \left(0.615<x<1\right) \end{array}\right.$$

The cownose ray’s forward momentum comes mainly from its broad pectoral fins. Taking the pectoral fin plane as the reference, the deformation generated by the pectoral fin motion can be divided into upward and downward slapping strokes. The upward and downward oscillations are made by the anterior side of the pectoral fins first, and then they extend to the tip and posterior side of the fins. The overall motion can be approximated as a traveling wave passing from the head to the tail [21]. The motion of the pectoral fin deformation was analyzed with reference to the motion morphology of the cownose ray, assuming the same amplitude for its up and down oscillation stroke. The direction along the chord is a traveling wave passing from the head to the tail. Along the spreading direction, the amplitude of pectoral fin deformation varies with the y-coordinate, and the amplitude is larger closer to the edge of the pectoral fin. The variation curve of amplitude with the y-coordinate essentially conforms to the quadratic curve, so the variation law is fitted with a quadratic polynomial. Combining the existing studies, the deformation produced by the pectoral fin can be represented by the following model [14,21]:

(2) $$\left\{\begin{array}{l} z\left(x,t\right)=A(y)\sin(\omega t-kx)\\ k=\frac{2\pi }{\lambda }\\ \omega =2\pi f_{0}\\ A(y)=ay^{2}+by \end{array}\right.$$

where *x, y, z* are the three-dimensional coordinates of the corresponding points on the pectoral fin coordinate system of the cownose ray. *t* is the time of motion. λ is the wavelength of the traveling wave of the pectoral fin. *f_0_* is the pectoral fin upwelling frequency. *A(y)* is the second-order amplitude expansion factor along the spreading direction. *a*, *b* are the amplitude expansion factor coefficients.

From the above model, it can be seen that the displacement in the plumb direction of any point on the pectoral fin of the cownose ray is related to the chordwise and spreading coordinates as well as the wavelength and frequency of the traveling waves. Therefore, the amplitude, number of waves, and fluctuation frequency of the traveling waves can be adjusted to change the whole pectoral fin fluctuation deformation pattern.

### 2.2. Structural Design of the Secondary Configuration of the Caranx melampygus

#### 2.2.1. Biological Characteristics of the *Caranx melampygus*

The carangidae belongs to the order Perciformes of the Scleractiniaceae. The fish body is prolonged and laterally flattened, with an oval or rhombic shape and a thin tailstock. The *Caranx melampygus* has a small width of carapace spread and the caudal fin is fork-shaped [22]. Its propulsion mode belongs to the BCF mode, which enables fast swimming [23]. The secondary configuration of the robotic fish developed in this study takes the *Caranx melampygus* with tail swing as the propulsion mode as a reference for the design of its tail fin structure.

#### 2.2.2. Physical Model and Motion Analysis of the *Caranx melampygus*

**Figure 2:**
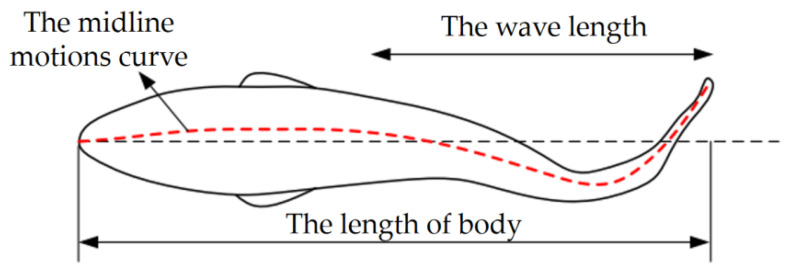
Schematic diagram of BCF fish midline motion curve.

When the bionic fish is in the secondary configuration, the shape of the entire fish body is determined by the size of the main configuration and the variation of the pectoral fins. The drive is achieved by the swing of the tail fin. Thus, the model and movement of the tail fin were mainly analyzed. The shape of the tail fin was designed with reference to the *Caranx melampygus* as a forked shape with similar top and bottom morphology, and the exact size depended on the height of the entire body in the secondary-configuration state. For the BCF mode, the fish swimming process can be analyzed using a viscoelastic beam model. The fish body is considered as a flexible viscoelastic beam and its interaction with the fluid is considered. The motion characteristics are analyzed using the midline motion curve as shown in the following equations [24,25]. The schematic diagram of the midline motion curve of the BCF fish is shown in Figure 2.

(3) h(x′,t)=H(x′)sin(wt−k′x′)

(4) $$H(x\prime )=a_{1}+a_{2}x\prime +a_{3}x\prime^{2}$$

where *H(x*′) is the fish envelope equation. *a*_1_*, a*_2_*,* and *a*_3_ are the envelope coefficients. *k*′ is the wave number of the fish wave. w is the frequency of the tail fin oscillation. *x*′ is the axial displacement of the fish body (head to tail direction). *t* is the motion time.

For BCF, fish swim by bending their body to change the pressure distribution of the surrounding fluid. The main influences on the swimming characteristics of fish waves are oscillation frequency, fish envelope, and fish wave number [26,27]. In the case of carangidae, *a_1_, a_2_, a_3_,* and *k′* in the equation are parameters related to the length of the fish. The control of the swimming situation of the secondary configuration can be achieved by changing the frequency and amplitude of the tail fin oscillation.

### 2.3. Overall Design of Variable-Configuration Bionic Robotic Fish

#### 2.3.1. Overall Design of the Structure

**Figure 3:**
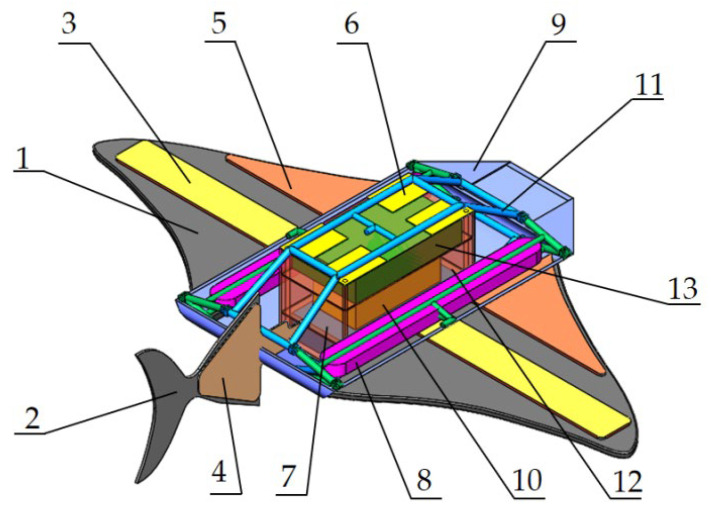
Overall structure of the robotic fish: 1—pectoral fins; 2—tail fin; 3—variable-configuration actuators (pectoral fins); 4—tail fin motion actuator; 5—pectoral fin motion actuator; 6—variable-configuration actuation airbag (fish body); 7—battery pack; 8—counterweight blocks; 9—shell; 10—pneumatic valves; 11—flexible internal bracket; 12—waterproof sealing box; 13—circuit board.

The overall structure of the bionic robotic fish is designed according to the characteristics and models of the main- and secondary-configuration bionic objects. The structure of the robotic fish in the main configuration is shown in Figure 3. It includes three major parts: the fish body, pectoral fin, and caudal fin. This prototype is pneumatically driven.

The fish body part includes a housing, a flexible internal bracket, the variable-configuration actuation airbags, a main control circuit board, a solenoid valve set, a battery, and a counterweight block. The shell is made of thermoplastic polyurethane elastomer rubber (TPU) material. The flexible internal bracket has a certain stiffness when inflated to support the flexible shell. The flexible internal bracket and two sets of variable-configuration actuators form the variable-configuration module. The pneumatic valve module, sensor module, battery module, and control circuit board are fixed in the middle of the pneumatic bracket and sealed with water resistance to balance the structural center of gravity of the bionic robot. The center of gravity and the floating center of the machine fish are on the chordal centerline of the fish body, and mean the center of gravity is located directly below the floating center. This can improve the stability of the machine fish in the water, preventing the occurrence of rollover. The upper surface of the shell has a floating and sinking control airbag, which works together with the counterweight block to control the floating and sinking state of the machine fish. Both the pectoral and tail fins are actuated by pneumatic flexible bending actuators. In order to achieve a good flexible deformation of the pectoral and caudal fins to complete the oscillating propulsion, soft and flexible materials were used to make the fins.

#### 2.3.2. Overall Design of the Structure

**Figure 4:**
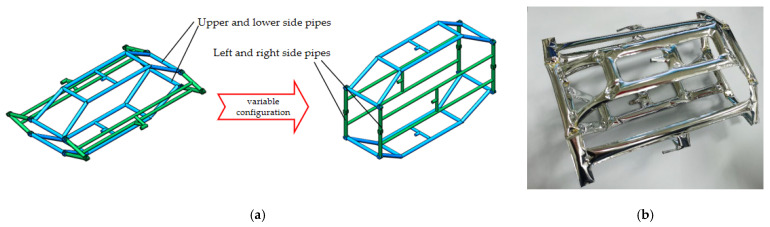
Inflatable flexible bracket: (**a**) three-dimensional model of the main and secondary configurations; (**b**) physical drawing of the bracket.

The variable-configuration module consists of a flexible internal bracket and two sets of variable-configuration actuators. An inflatable flexible internal bracket was designed to enable the variation between the main and secondary configurations of the machine fish. The bracket is made of aluminized polyester. It consists of the same upper and lower side pipes and the same left and right side pipes in four parts. Two sheets of aluminum plastic film were laser cut on each side of the piping according to the profile of the inflatable bracket and welded together with the edges. Bending is achieved by intermediate spot welding and connected by Kevlar wire to form a three-dimensional support. The inflatable flexible bracket is shown in Figure 4. Inflating the inside of the pipes provides stiffness to the bracket. It supports the flexible shell and fixes the internal module to ensure the stability of the robotic fish when it swims underwater. On the other hand, due to the compressibility of the gas, the bracket has a certain degree of elasticity so that the machine fish has a certain anti-vibration ability.

**Figure 5:**
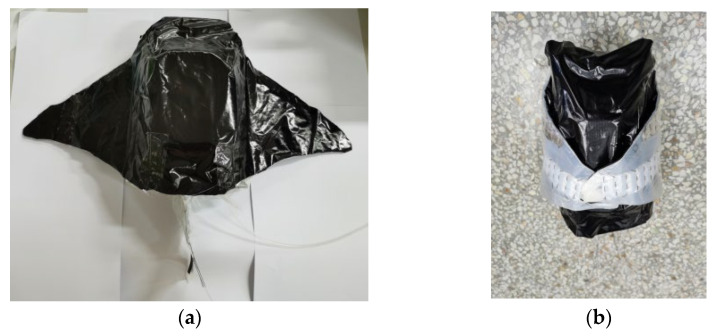
Physical diagram of variable-configuration machine fish: (**a**) main configuration; (**b**) secondary configuration.

Variable-configuration actuators include fish body variable-configuration airbags and pectoral fin variable-configuration actuators. There are four fish variant airbags, each of which is a corrugated tubular airbag made of aluminum plastic film, symmetrically distributed above the waterproof sealing box. The two ends of the airbag are fixed to the waterproof sealing box and the internal bracket, respectively. The machine fish’s center of gravity remains below the prototype after it completes the variable configuration. This improves its stability when swimming underwater. When executing the command to change from the main configuration to the secondary configuration, the variable-configuration airbag is inflated and raised, lifting the top of the internal bracket and deforming the bracket. A pneumatic flexible actuator is provided in the middle of the upper surface of each pectoral fin on both sides. The input values for the pectoral fin variable actuator are based on the value of the change in the height of the fish. The actuators are inflated and bent upward when executing the conformation command, and the pectoral fins are lifted upward and attached to the upper surface of the fish body. The shape of the robotic fish changes from horizontal flat to lateral flat. When the secondary configuration changes to the primary configuration, the variable-configuration airbag is deflated and the airbag returns to its original shape under the action of elastic rubber. The internal bracket is dropped and the pectoral fin actuator is deflated. The width is 50 cm and the height is 9 cm for the main configuration of the machine fish; the width becomes 12 cm and the height becomes 16 cm for the secondary configuration. The width reduction rate after the conformation change is 76% and the height increases by 33%. The prototype of the variable-configuration machine fish is shown in Figure 5.

#### 2.3.3. Design of the Drive Module

The drive module mainly contains the pectoral fin drive module for the primary configuration and the tail fin drive module for the secondary configuration. The drive actuator used is a laboratory-developed pneumatic flexible actuator. The actuator bends and deforms when ventilated, and the degree of bending is influenced by the internal air pressure. The fins were made from silicone sheets by laser cutting. In order to achieve a good oscillation effect, elastic PET plate reinforcements were added at the end of the pectoral and tail fin actuators.

**Figure 6:**
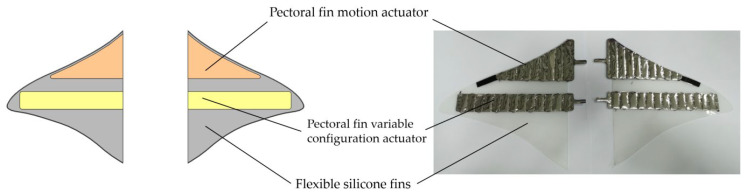
Pectoral fin drive module and its physical object.

The pectoral fin drive module consists of flexible fins and actuators. The shape of the flexible fins was obtained by extracting the pectoral fin profile in Section 2.1. An actuator was distributed on each side of the fins in front of each pectoral fin. The pectoral fins bend upward when the upper surface motion actuator is inflated and bend downward when the lower surface actuator is inflated. This enables bi-directional bending of the fins. During motion, the two motion actuators on the upper and lower surfaces of each fin are alternately inflated and bent to periodically drive the front part of the fins, achieving an overall up-and-down slap of the pectoral fins to generate forward thrust. Providing different drive air pressures or frequencies to the two pectoral fins will cause asymmetric thrusts on both sides of the pectoral fins as a way to achieve directional control of the robotic fish. The pectoral fin drive module and its physical appearance are shown in Figure 6.

**Figure 7:**
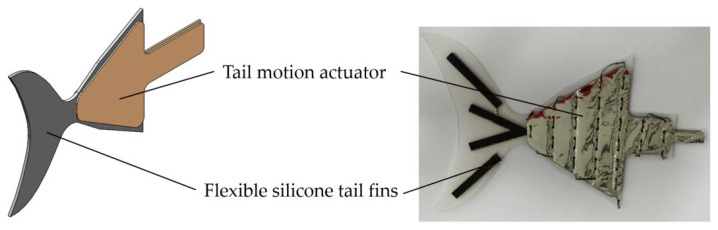
Tail fin drive module and its physical object.

The tail fin drive module consists of the flexible fin and the actuator. The contour structure of the flexible fin was obtained by extracting the tail of the *Caranx melampygus*. The two actuators are located on the two sides of the fins on the front section of the tail fin. The tail fin bends to the left when the left motion actuator is inflated and to the right when the right motion actuator is inflated. By alternately inflating the two actuators periodically, the front section of the tail oscillates from side to side. The posterior portion of the caudal fin is the passive part, which is influenced by the front section then swings to produce a forward-to-backward traveling wave that propels the robotic fish forward. The method of its direction control is similar to the pectoral fin drive. Different driving air pressures or frequencies are supplied to the actuators on each side of the tail fin, causing the tail fin to swing asymmetrically to both sides, generating asymmetrical thrust. The tail fin drive module and its physical appearance are shown in Figure 7.

## 3. Design of Motion Control System for Variable-Configuration Robotic Fish

### 3.1. Overall Solution and Design of the Control System

**Figure 8:**
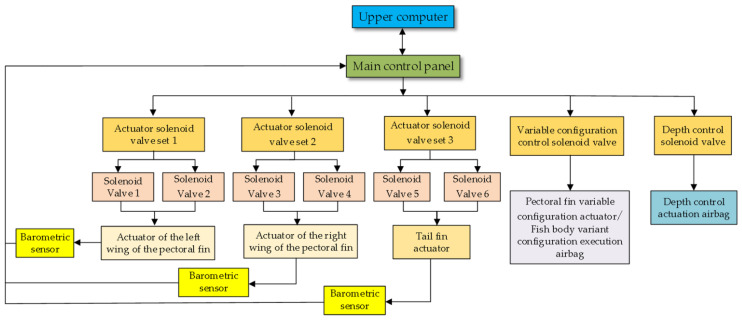
Overall solution and design of the control system.

Based on the design of the structure of the variable-configuration bionic robotic fish, it was determined that the flexible pneumatic bending actuator is used to realize the movement of the pectoral and tail fins, and the variable configuration of the torso is realized by using the actuating airbag. The purpose of adapting to various swimming environments with different configurations is achieved. The overall design of the robotic fish control system should be from the aspect of the gas drive, as shown in Figure 8.

The main control board in the lower computer controls the pneumatic actuators through multiple solenoid valve sets and collects the air pressure data from the corresponding actuators through air pressure sensors. Bluetooth serial communication is used between the main control board and the upper computer to send the collected air pressure data to the upper computer and receive the control commands from the upper computer. The pneumatic actuation module mainly consists of the air source, pressure reducing valve, solenoid valve set, and pneumatic actuator. The pneumatic actuators include two sets of left and right pectoral fin motion actuators, one set of tail motion actuators, an actuating airbag to control the variable configuration of the body, the pectoral fin variable-configuration actuators, and an actuating airbag to control the depth. The pectoral fin and tail fin motion actuators are controlled by two air circuits to achieve bi-directional fin oscillation.

The hardware part of the machine fish mainly includes the PC, main control circuit board, amplifier circuit, solenoid valve group, air pressure sensor, air source, and pressure-reducing valve. The main control board circuit outputs PWM waves to control the amplification circuit. The solenoid valve set is connected to the amplifying circuit, and the gas from the gas source reaches the solenoid valve set through the pressure-reducing valve. The air pressure sensor data are collected by the main control board after AD conversion. The hardware circuit mainly realizes the tasks of voltage conversion, solenoid valve control, data acquisition, and communication with the upper computer. The voltage provided by the lithium battery is reduced by the step-down voltage regulator chip to achieve power supply to each module as well as the MCU. The STM32 is used as the main control chip, and the data are transmitted through the serial port.

The software system of the machine fish is divided into the lower computer (main control board) software system and the upper computer software system. The lower computer is responsible for receiving commands from the upper computer. According to the instruction, it controls the corresponding solenoid valve action to realize the target action of the machine fish, and at the same time collects the air pressure data of the motion actuator and sends the data to the upper computer in real time. The upper computer is responsible for sending motion mode or motion parameter commands to the lower computer to change the motion state of the machine fish. It receives the corresponding movement solenoid valve air pressure sensor data from the lower computer and records them to determine the real-time change in air pressure and monitor the working status of the machine fish.

### 3.2. Motion Control of Variable-Configuration Robotic Fish

**Figure 9:**
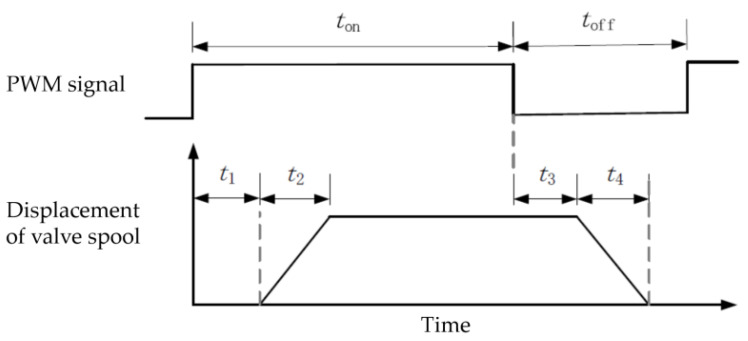
Relationship between PWM signal and spool motion.

The dynamic control of air pressure in the pneumatic system is achieved through the filling and deflating of high-speed switch valves. The operating principle of high-speed switch valves is to use the electromagnetic force to open the spool after the power is applied, and the spool is closed under the action of spring force after the power is disconnected, which can realize the fast switching of the spool state. In order to realize the control of the high-speed switch valve to drive the machine fish swimming with the help of the PWM signal, the relationship between the PWM signal and the spool displacement change needs to be analyzed. When a high-speed switch valve is driven using a PWM signal, the switch valve is energized for a time $t_{on}=r/f_{PWM}$ and de-energized for a time $t_{off}=\left(1-r\right)/f_{PWM}$ during one signal period, where $f_{PWM}$ is the frequency of the PWM signal and *r* is the duty cycle. After the high-speed switch valve is energized, the coil generates a magnetic field, the core is magnetized, and the electromagnetic force on the spool gradually increases until it equals the spring force, which is recorded as *t*_1_. Then, the spool starts to move, and the time required from the start of the movement of the spool to the completion of the movement is recorded as *t*_2_. When the switching valve is de-energized, the core is demagnetized, and the electromagnetic force on the spool decreases until it equals the spring force, which is recorded as *t*_3_. When the electromagnetic force is less than the spring force, the spool starts to reset, and the time required for the spool to reset from the beginning to the end is recorded as *t*_4_. The process is shown in Figure 9. In order for the spool of the high-speed switch valve to respond to the PWM signal, *t_on_* > *t*_1_ and *t_off_* > *t*_3_ is required. Although increasing the PWM frequency can reduce the response variation value, it is not conducive to duty cycle regulation [28]. We consulted the operating manual of the switching solenoid valve to determine *t_on_* = 3.5 ms, *t_off_* = 2 ms. The operating frequency of 50 Hz was chosen to drive the switching solenoid valve after comprehensive consideration.

**Figure 10:**
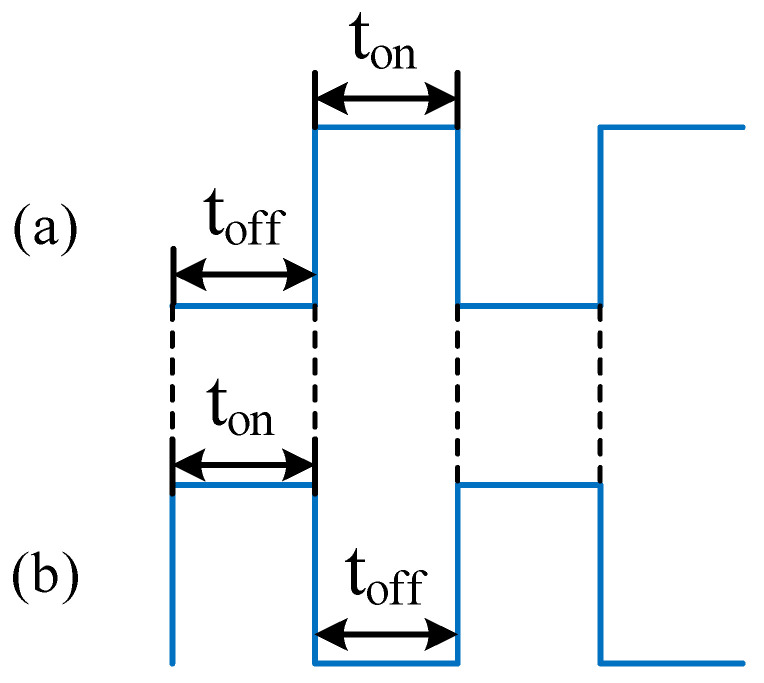
Start–stop time sequences of the PWM wave. (**a**) The upper surface actuator of the left pectoral fin during advancement; (**b**) The lower surface actuator of the left pectoral fin during advancement.

In the main configuration, the leading edge of the pectoral fin is controlled to slap up and down by feeding a PWM wave to the solenoid valve set of the pectoral fin motion actuator. The start–stop time sequence of the PWM wave during this process is shown in Figure 10a,b, indicating the control solenoids for the left pectoral fin upper and lower surface motion actuators, respectively. The input PWM waves of the upper and lower surface actuator solenoids are exactly opposite, driving the pectoral fins to perform alternating upward and downward slapping periodically. The secondary configuration is propelled by tail fin oscillation. There is a pneumatic actuator on each side of the tail fin, and the opposite PWM wave is input to the control solenoid valve on both sides to make the tail fin swing alternately from left to right periodically.

The speed control of the machine fish depends on several parameters, such as oscillation frequency, oscillation amplitude, and oscillation section length [29], while the speed of the machine fish is also influenced by the thrust gradient, thrust delay, and drag [30]. In this paper, the amplitude and frequency of movement of the pectoral or tail fin were changed by controlling the driving air pressure and driving frequency of the actuator, both of which are related to the duty cycle of the PWM wave. Therefore, speed control of the machine fish was achieved by changing the duty cycle and start–stop frequency when the PWM wave was turned on.

The robotic fish’s direction also needs to be controlled when it is moving underwater. In the main configuration, the duty cycle of the PWM waves driven by the pectoral fins on both sides was changed so that the air pressure inside the pectoral fin actuators on both sides was different. The different slap of the pectoral fins changed the swimming direction of the robotic fish. In the same way, the steering action was achieved by changing the swing amplitude of the actuators on both sides of the tail fin of the secondary configuration.

## 4. Underwater Experiments and Discussion of Variable-Configuration Robotic Fish

### 4.1. Construction of the Underwater Test Platform

**Figure 11:**
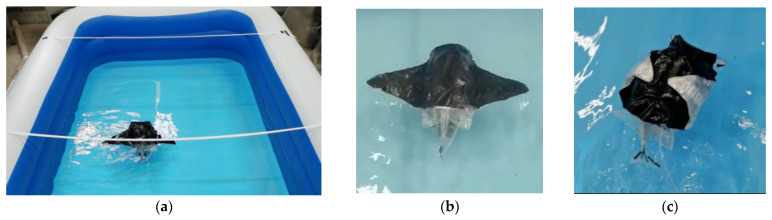
Underwater test of variable-configuration bionic robotic fish: (**a**) underwater test bench; (**b**) the swimming state of the main configuration; (**c**) the swimming state of the secondary configuration.

After completing the design of the structure and control system of the machine fish, a set of prototype machines were trial-produced. The experimental study of the propulsion performance of the machine fish was conducted after the commissioning of the underwater environment operation to ensure that the prototype could work properly. The underwater test bench mainly included the bionic robotic fish prototype, an inflatable swimming pool, camera, air compressor, and computer. An area of 1 m × 1.4 m near the middle of the pool was selected as the experimental data collection area, and two lines were set as the location marker lines. An external air compressor was used as the air source for the machine fish. A PC was used as the upper computer to communicate with the machine fish through a Bluetooth module. The camera was fixed in the data acquisition area to record the motion status of the machine fish during the experiment, and the video was processed to be able to obtain the motion performance parameters. The test bench and the process of the robotic fish swimming underwater are shown in Figure 11.

The test was conducted using floating and sinking control to keep the machine fish mostly submerged. The robotic fish started swimming from one end of the pool and the time it took to pass through the data collection area was recorded. The average propulsion speed of the robotic fish was derived by recording the time it took to pass a fixed distance. The speed variation law of the robotic fish under different motion parameters was obtained by changing the frequency and amplitude of the fins. The magnitude of air pressure in the actuator was changed by controlling the duty cycle of the PWM wave of the high-speed switching valve to control the amplitude of the pectoral and caudal fins during the test, and the pressure inside the pneumatic actuator was monitored in real time.

### 4.2. Underwater Performance Test of the Main Configuration of the Cownose Ray

#### 4.2.1. Straight Line Propulsion Test

**Figure 12:**
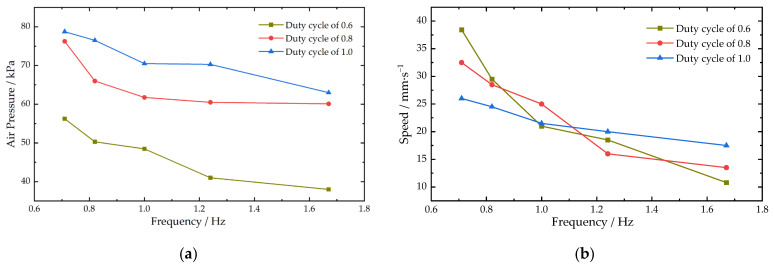
Main-configuration robotic fish underwater linear propulsion test results: (**a**) changes in peak air pressure in the pectoral fin actuator; (**b**) changes in the average speed of linear propulsion.

It has been shown that the fin slapping frequency of cownose rays is low, averaging about 1.0 Hz, with an amplitude index of about 0.35, a wave number of about 0.4, and a forward distance of about 60 cm in one cycle of pectoral fin movement [19]. Referring to the motion parameters of real cownose rays, different oscillation frequencies and amplitudes were set for the experiments of the propulsion speed of the main-configuration robotic fish. The frequency was controlled by controlling the period of the pectoral fin slap, which is the time between one upward and one downward slap of the pectoral fin, and setting the period from 0.6 s to 1.4 s, corresponding to a frequency of 1.67 Hz to 0.71 Hz. The PWM wave duty cycle was set to 0.6, 0.8, and 1 to obtain the propulsion speed variation of the main configuration of the machine fish at different frequencies. The average swimming speed and the variation in air pressure in the actuator of the main configuration of the robotic fish for different duty cycles and frequencies are shown in Figure 12.

The results showed that the peak air pressure in the actuator decreased with increasing frequency, and the overall trend was similar for different duty cycle conditions. The duty cycle of the high-speed switch valve PWM wave was the main factor affecting the pressure inside the actuator, and the larger the duty cycle, the greater the pressure. The maximum pressure inside the actuator was 78.75 kPa. The ranges of the average speed of pectoral fin slap propulsion were 10.80~38.42 mm/s, 13.49~32.45 mm/s, and 17.61~26.09 mm/s when the duty cycle was 0.6, 0.8, and 1, respectively. The higher the duty cycle, the smaller the range of the average speed. The speed decreased less with increasing frequency in large duty cycles.

#### 4.2.2. Turning Performance Test

**Figure 13:**
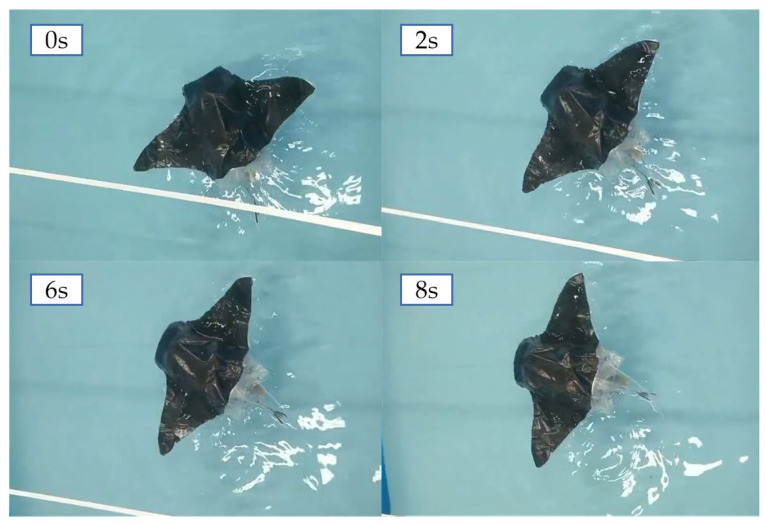
Turning process of the main configuration of the machine fish.

In the experiments of the turning performance of the main-configuration robotic fish, the underwater turning was accomplished by controlling the asymmetric slap of both pectoral fins. Based on the results of the main-configuration propulsion speed experiments, the pectoral fins on one side were made to adopt the oscillation frequency and amplitude of the minimum propulsion speed, and the pectoral fins on the other side were moved with the oscillation frequency and amplitude of the maximum propulsion speed. The underwater swimming process of the robotic fish was captured with a camera, and the speed and turning radius of the robotic fish’s turns were obtained by processing the video offline. The turning process in the main-configuration state of the machine fish is shown in Figure 13, and the angular speed of the turn was calculated to be 5.6°/s and the turning radius was 36 cm.

### 4.3. Underwater Performance Tests of the Secondary Configuration of the Caranx melampygus

#### 4.3.1. Straight Line Propulsion Test

**Figure 14:**
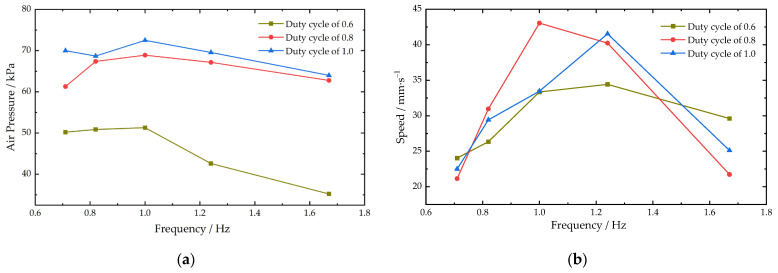
Secondary-configuration robotic fish underwater linear propulsion test results: (**a**) changes in peak air pressure in the tail fin actuator; (**b**) changes in the average speed of linear propulsion.

By sending commands to the robotic fish, the body was changed from horizontal flat to lateral flat and the pectoral fins were bent upward. The change from the main configuration of the cownose ray to the secondary configuration of the *Caranx melampygus* was completed. The tail fin propulsion command allowed the robotic fish to swim using a tail fin swing. The time of the robotic fish passing through the two marker lines in the data collection area was recorded to obtain the average speed of propulsion of the robotic fish in the secondary-configuration state. The PWM wave duty cycle was set to 0.6, 0.8, and 1, and the pressure inside the tail fin actuator was monitored in real time to test the average propulsion speed at different frequencies of the secondary configuration. The average swimming speed and the variation of air pressure in the tail fin actuator for different duty cycles and frequencies are shown in Figure 14.

The peak air pressure in the tail fin actuator affected by duty cycle and frequency was similar to the results for the pectoral fin. The air pressure increased more when the duty cycle increased from 0.6 to 0.8 and less when the duty cycle exceeded 0.8. The maximum pressure in the actuator was 72.50 kPa. The ranges of the average tail fin swing propulsion velocity were 26.37–34.04 mm/s, 22.51–41.54 mm/s, and 21.04–43.05 mm/s when the duty cycle was 0.6, 0.8, and 1, respectively. The speed tended to increase and then decrease with increasing frequency, and the change was more obvious when the duty cycle was larger. The speed peaks for different duty cycles all appeared around the frequency range of 1 to 1.2 Hz.

#### 4.3.2. Turning Performance Test

**Figure 15:**
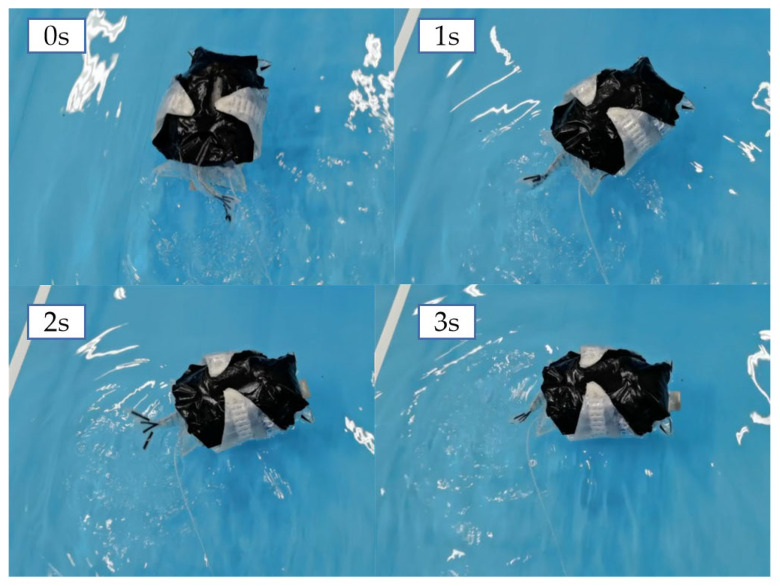
Turning process of the secondary configuration of the machine fish.

The directional control of the secondary-configuration robotic fish was achieved by the left–right asymmetric oscillation of the tail fin. Based on the experimental results of the propulsion speed of the secondary configuration, the motion frequency and amplitude of the maximum propulsion speed were selected to control the actuating airbag on the tail fin side, and the actuator on the other side was controlled using the motion frequency and amplitude of the minimum propulsion speed. The turning process of the machine fish in the secondary-configuration state is shown in Figure 15. The captured video images were processed and analyzed to obtain its turning angle speed of 30°/s and turning radius of 15 cm.

### 4.4. Experiment Summary and Discussion

According to the two driving methods of this variable-configuration bionic fish, the effects of fin movement frequency and amplitude on the propulsion speed of the two configurations were investigated. In addition, the motion control parameters under the maximum propulsion speed were selected to test the turning speed and turning radius of the machine fish in the two configurations. The experimental results are summarized in Table 1.

The main configuration of the machine fish propulsion speed is influenced by the frequency of the pectoral fin motion, and the duty cycle has relatively little effect on the machine fish propulsion speed. As the frequency increases, the propulsion speed of the machine fish decreases, as shown in Figure 12b. This is due to the increase in the speed of fin oscillation to increase its resistance in the water, resulting in a decrease in fin oscillation and a small drainage volume, so the propulsion speed becomes smaller. The effect of frequency on speed decreases as the duty cycle increases. The reason is that a larger duty cycle provides a higher pressure to the actuator, which increases the actuator bending amplitude. At higher frequencies, the increase in duty cycle increases the amplitude of the pectoral fin oscillation. This improves the propulsion speed, which is similar to the results of the literature [19,20]. At lower frequencies, the machine fish with a small duty cycle is propelled at a greater speed. This is due to the fact that this frequency is the commutation frequency of the pectoral fin oscillation. When the frequency is small, the pectoral fin oscillates to one side for a longer period of time, and the pectoral fin oscillates to the limit position for a shorter period of time under large duty cycle conditions, so the pectoral fin will hover at the highest and lowest points, resulting in a reduced speed.

The average velocity of the tail fin propulsion of the robotic fish secondary configuration increases and then decreases with increasing frequency, as shown in Figure 14b. When the frequency increases in the low-level range, the number of tail fin oscillations increases, which will increase the stroke per unit time to improve the propulsion speed. This result is similar to the findings of the literature [31]. When the frequency exceeds 1.2 Hz, the speed of fin oscillation will increase its resistance in the water due to the speed of fin oscillation. This leads to a decrease in the amplitude of fin oscillation and a decrease in speed. The inadequate bending of the left and right actuators of the fins also increases the unevenness of the tail fin swing. Therefore, at higher frequencies, the robotic fish’s secondary-configuration propulsion is prone to yaw, which needs to be compensated for and controlled by adjusting the duty cycle. The robotic fish imitating the *Caranx melampygus* in the secondary-configuration state had higher speed than in the main configuration of the cownose ray, and the main configuration swam more smoothly and with less disturbance to the water environment, which essentially matched the movement state of the real fish [32]. Through the above experiments, it was found that the machine fish secondary configuration can achieve a larger angular speed and smaller turning radius; this result corresponds to the secondary configuration having a larger propulsion speed, while the robotic fish main configuration has the advantage of smaller perturbation in motion.

In this study, we designed a bionic fish with variable configurations, using cownose rays and *Caranx melampygus* as the main and secondary configurations, respectively, and conducted theoretical analysis and research on the propulsion of the two configurations. On this basis, an experimental study of its underwater propulsion performance was conducted to verify the feasibility of the structural design and control methods, and the results can be used as a reliable basis for subsequent simulations and mechanism optimization studies for this machine fish. This study can provide some reference for the design of variable-configuration bionic robotic fish, but there are still some limitations. The structure was not designed with a built-in air source pump, but with an external air compressor to provide power, limiting the underwater swimming range and portability of the bionic robotic fish. Follow-up studies may consider the use of a built-in gas source pump, or the design of a gas circulation loop. The designed pectoral fin propulsion mode is only driven periodically by a set of actuators at the front of the pectoral fin. The method is simple in structure and control and achieves good propulsion performance, but there is still a gap with respect to the actual motion of the bionic object. To further investigate the effect of the pectoral fin oscillation condition on the propulsion performance, multiple sets of actuators should be arranged in the pectoral fin. In this study, only the air pressure sensor is used to monitor and regulate the swimming of the robotic fish. We may consider adding other sensors and optimizing the control method to achieve closed-loop control to improve the underwater swimming performance of the robotic fish as well as its autonomous swimming.

## 5. Conclusions

An experimental prototype of a pneumatic variable-configuration machine fish was developed in this paper. Analysis and structural design were carried out based on the characteristics possessed by the bionic object, and a variant configuration scheme was determined. The designed robotic fish can achieve the pectoral fin oscillation propulsion of the main configuration of the cownose ray and the tail fin oscillation propulsion of the secondary configuration of the *Caranx melampygus*. The shape of the pectoral fin and the fish body can be changed smoothly to complete the variation in configuration. For the characteristics of the pneumatic drive, a hardware and control circuit and pneumatic platform were designed, and the upper and lower computer and drive control strategy were determined. The propulsion performance of the primary and secondary configurations was investigated through underwater tests. The maximum propulsion speed of the main configuration of the machine fish was 38.24 mm/s, the turning angle speed was 5.6°/s, and the turning radius was 360 mm. The maximum propulsion speed in its secondary configuration was 43.05 mm/s, the turning angle speed was 30°/s, and the turning radius was 15 mm. The test results verified the feasibility of the structure design and propulsion mode of the robotic fish, and provided reliable support for subsequent research on the simulation and optimization design of the robotic fish.

## Figures and Tables

**Table 1:** Performance parameters of robotic fish main configuration and secondary configuration.

Parameters	Main Configuration	Secondary Configuration
Width of the body plate	50 cm	12 cm
Height of the body plate	9 cm	16 cm
Maximum propulsion speed	38.42 mm/s	43.05 mm/s
Turning angle speed	5.6°/s	30°/s
Turning radius	36 cm	15 cm

## Data Availability

The data collected in this research are available upon request.
